# Examining Undergraduates’ Intentions to Pursue a Science Career: A Longitudinal Study of a National Biomedical Training Initiative

**DOI:** 10.3390/educsci15070825

**Published:** 2025-06-28

**Authors:** Jayashri Srinivasan, Krystle P. Cobian, Hector V. Ramos, Christina A. Christie, Catherine M. Crespi, Teresa Seeman

**Affiliations:** 1 School of Education and Information Studies, University of California, Los Angeles, CA 90095, USA; 2 Geffen School of Medicine, University of California, Los Angeles, CA 90095, USA; 3 Department of Biostatistics, Fielding School of Public Health, University of California, Los Angeles, CA 90095, USA; 4 Departments of Medicine and Epidemiology, University of California, Los Angeles, CA 90095, USA

**Keywords:** STEM program evaluation, career intentions, longitudinal study, propensity score estimation, undergraduate research experiences

## Abstract

Disparities in the participation of individuals from historically excluded groups in science careers persist, particularly at advanced career stages. In response to this challenge, the National Institutes of Health developed the BUilding Infrastructure Leading to Diversity (BUILD) initiative, aimed at undergraduate institutions to examine evidence-based strategies to engage and retain students across science-related fields. In this longitudinal study, we used propensity score matching and mixed-effects logistic regression models to examine the effects of BUILD on undergraduates’ intentions to pursue science-related research careers. The results indicate that students who participated in BUILD are four times more likely to pursue a science-related research career in comparison to their non-BUILD counterparts. We also discuss and present the need to incorporate research training and mentorship to promote a diverse scientific workforce.

## 1. Introduction

The proportion of individuals from underrepresented groups entering science-related careers has remained low in comparison to the increasingly diverse population across the United States (Fry et al., 2021; Hurtado et al., 2017; National Science Board, 2021). These disparities are persistent across the growing U.S. population at all educational and career stages of the scientific workforce (Chen et al., 2022; Hurtado et al., 2017; Saw et al., 2018). In response to national calls to reduce disparities in participation in the science, technology, engineering, mathematics, and medicine (STEMM) workforce, federal and private funders have invested in programs and research to better understand and address disparities in STEMM training and workforce participation (James & Singer, 2016). To better support efforts to promote workforce diversity, it is vital to track students throughout their time in college about their psychological and academic experiences, along with gathering stronger empirical evidence regarding what programs work to increase interest and persistence for undergraduates toward a STEMM career path.

Informed by these needs, in 2014 the National Institutes of Health (NIH) made a historic and sizable 10-year investment to form the Diversity Program Consortium (DPC), managed by the National Institute of General Medical Sciences (NIGMS), that aims to develop, implement, assess, and disseminate innovate approaches to research training and mentoring. One of DPC’s largest research training and institutional-capacity-building initiatives is the BUilding Infrastructure Leading to Diversity (BUILD) initiative (Gibbs et al., 2022). BUILD programs, implemented at multiple institutions in the U.S., provide a range of student and faculty-focused activities and funding, including but not limited to faculty development, increased institutional research capacity, and increased commitment to engaging and retaining students from diverse backgrounds in the biomedical research workforce (Davidson et al., 2017).

To address the gaps in our understanding of what interventions work for students from diverse backgrounds, the broader BUILD evaluation followed undergraduate students at the BUILD institutions and studied key psychosocial and academic outcomes, such as science identity, intentions to pursue a science-related career, and enrollment in graduate school. BUILD institutions supported undergraduate students from all backgrounds by mentoring and training them as they pursued their degree, to enhance the long-term goal of how to best prepare researchers from diverse backgrounds to become contributors to the STEMM research community. The purpose of this study is to examine undergraduate students’ intentions to pursue a science-related research career, which serves as an important signal of students’ intentions to stay in academia (or research-related careers), and is also a measure of their long-term readiness to be a part of the academic social system, including conducting research, applying to graduate school, and attending graduate school (Estrada et al., 2011). This study adds to the literature on STEMM in two important ways. First, because research suggests that undergraduate students’ science-related research career intentions decrease over time (Schultz et al., 2011), our study uses longitudinal data of four cohorts (Fall 2016, 2017, 2018, and 2019) of undergraduate students who were enrolled as freshmen and were then tracked annually through 2022. This enables us to examine any change in students’ intentions regarding pursuit of a science-related research career over time, thus accounting for students’ maturation over academic years, and not just a snapshot of their intentions at a single time point. Second, this longitudinal study documents the progress of students who participated in the BUILD program compared to those who did not and makes use of a propensity score matching approach to control for selection bias with respect to BUILD participation. This analytic approach of employing a comparison group strengthens the inferences we can make about the students who participated in the BUILD program and their intentions to pursue a science-related research career. These design aspects of our study facilitate the identification of the key changes needed to support the evolution of the scientific “thinkforce” to better represent the demographics of the US population (Davidson et al., 2017). We address the following research questions:

1. Does participation in the BUILD program impact undergraduate students’ intentions to pursue a science-related research career over time?
2. Do the key BUILD initiative components of research experience and mentorship contribute to any observed differences between intentions to pursue a science-related research career for BUILD and non-BUILD students?

### 1.1. The BUilding Infrastructure Leading to Diversity (BUILD) Initiative

The BUILD program consisted of 10 NIH-funded awards to 11 higher education institutions (one award was shared by two institutions) geographically dispersed across the United States. Institutional eligibility for BUILD awards included having fewer than $7.5 million in total NIH research project grant funding and a student population with at least 25% Pell Grant recipients. Thus, this study had the opportunity to focus on students at institutions with highly diverse student populations and examine intentions to pursue a science-related research career at institutions with relatively less research capacity compared to institutions with high research activity and a history of NIH research project grant funding. In the current study, about 43% of students received Pell grants, and 26% were first-generation college students across these institutions. Additionally, BUILD awards support institutional development to sustainably enhance the training environment through physical renovations, purchasing state-of-the-art equipment, curricular redesigns, and developing partnerships with community colleges and high schools to help enhance their STEMM curricula and research opportunities.

The BUILD institutional programs were designed as comprehensive interventions composed of several standard components, such as research participation (Carpi et al., 2017; Chang et al., 2011; Eagan et al., 2013; Hurtado et al., 2009) and mentoring by both faculty and peers (Atkins et al., 2020), geared to successfully support students, including those from backgrounds that are historically marginalized, on a science-related career path. While there were shared grant requirements, each site was able to adapt student-level, faculty-level, and institutional-level activities to fit their institutional context and needs. For example, hours of undergraduate research requirements and/or the types of mentoring activities may have varied slightly between the sites (Cobian et al., 2024; Guerrero et al., 2022). The larger goal of the BUILD initiative was not only to establish effective programs that promote biomedical workforce diversity, but to include research and evaluation about why different programs are effective and to enable other colleges and universities to learn from the effort.

Each BUILD site provided interventions for students in both undergraduate research and mentoring (McCreath et al., 2017), while collaborating with the Coordination and Evaluation Center (CEC) to partner with BUILD sites to administer annual surveys to students, alumni, and faculty at each site, including those who were involved in BUILD programs and those who were not. In this study, we examined BUILD engagement as defined by students who received the most extensive BUILD exposure, i.e., the BUILD Scholars and BUILD Associates; see Maccalla et al. (2022, p. 61). BUILD Scholars often received tuition support or stipend, research training, and mentorship. Scholars were often required to participate in a host of BUILD-related activities. BUILD Associates often participated in a subset of BUILD-related activities but had fewer requirements than BUILD Scholars. At some sites, Associates went on to become Scholars; thus, we decided to include both groups in the study.

### 1.2. Undergraduate Research Experiences

The American Association of Colleges and Universities identifies Undergraduate Research Experiences (UREs) as a high-impact educational practice for achieving excellence in a science-related career (AAC&U, n.d.). Kuh and Kinzie (2018) elaborated on the phrase “high-impact practice” and discussed the need for assessing the efficacy of the implementation of these practices in higher education, specifically for students who are from historically underrepresented groups. UREs are a central component of STEMM interventions and are strongly associated with students’ intentions to pursue a science career (Hathaway et al., 2002; Robinson et al., 2018). In addition, UREs influence students’ interests in STEMM fields, contribute to their progression into STEMM graduate study (S. H. Russell et al., 2007), and impact their development of a science identity. For example, students who report having a higher science identity have higher ratings of identifying as scientists, often earn higher grades in science-related courses, and have higher chances of obtaining employment in a science-related field (Stets et al., 2017). Eagan et al. (2023) found that first-year students participating in BUILD programs had higher science identity scores than those who did not participate. A student’s science identity is developed through education, training, and hands-on experiences, and is also associated with interests in science careers (Linn et al., 2015).

Students who participated in undergraduate research have a better chance of being accepted into a science graduate program (Carter et al., 2009; Schultz et al., 2011; Eagan et al., 2013) and a longer period of engagement in research increases their chances of pursuing a career in science as well excelling in graduate school (S. H. Russell et al., 2007; Gilmore et al., 2015). For example, Maton et al. (2009) found that students involved in a science scholar program at the University of Maryland, Baltimore County were five times more likely to pursue a Ph.D. than the comparison students.

Current scholarship also identifies participation in undergraduate research training and faculty mentoring as essential to successful URE interventions (Eagan et al., 2023; Estrada et al., 2016; Ramos et al., 2023). Research participation has been shown to strongly predict academic and career outcomes like degree aspirations, interest in science, and science self-efficacy (Chang et al., 2011; Merolla & Serpe, 2013; L. Russell, 2007). Students who participate in research tend to have higher college GPAs, higher levels of intentions to pursue a science career, and higher chances of continued participation in research. They are also more likely to have a faculty mentor, increased academic achievement, and longer-term success. All these are key predictors for entry into STEMM graduate programs (Aikens et al., 2017; Atkins et al., 2020; Tsui, 2007; Winterer et al., 2020). Additionally, students who participate in STEMM research enrichment programs have higher chances of attending STEMM graduate programs. Because our study examines intentions to pursue a science-related research career, the results will add to the knowledge base on the connection between research participation and STEMM career trajectories.

### 1.3. Mentorship in STEMM

Receipt of mentorship in STEMM is associated with increased confidence in research skills, higher researcher self-efficacy, and science identity (A. M. Byars-Winston et al., 2015; Estrada et al., 2018), which increases STEMM persistence. In particular, mentorship that occurs within undergraduate research experiences successfully supports students with STEMM career aspirations (Linn et al., 2015). For example, students who receive less support from their mentors reported lower levels of self-confidence (Haeger & Fresquez, 2016). Because research participation and mentoring positively predict a host of STEMM outcomes, both were focal activities of the BUILD intervention. Every BUILD site incorporated a research and mentoring component into the intervention program, which further underscores the relevance of assessing both experiences in any STEMM intervention evaluation.

## 2. Materials and Methods

### 2.1. Data Source and Sample

For the current study, we employed a multi-site longitudinal survey data set collected across 11 higher education institutions. The data set for this study used three sources of survey data. First, the Higher Education Research Institute’s (HERI) Freshman Survey (TFS; Stolzenberg et al., 2020) was administered to incoming first-time, first-year students at the beginning of each fall semester; we employed a total of four cohorts starting Fall 2016 (Fall 2016, 2017, 2018, and 2019) across the 11 sites. The TFS asks about students’ pre-college attitudes, perceptions, and beliefs, and collects student demographic data as well. Second, the DPC’s Student Annual Follow-up Survey (SAFS; Eagan et al., 2023) was developed by the CEC, and these follow-up surveys were administered every spring from 2016 through 2022. The SAFS collects students’ perceptions and views on various educational and career goals, as well as experiences in college. The third source of data is the HERI’s College Senior Survey (CSS; Fregoso & Lopez, 2020), which all graduating seniors in the sample were invited to complete. Designed as an exit survey for graduating seniors, the CSS focuses on a broad range of college outcomes (e.g., academic achievement, satisfaction with college experience) and post-college goals and plans.

As a part of the broader study, BUILD and non-BUILD students were recruited and incentivized to participate in the study (see Ramirez et al., 2022 for more details). The overall response rates for the TFS administrations across the BUILD sites ranged from 40 to 60% each year (2016–2019), and once a student participated in the study, they were invited to each subsequent follow-up survey. There are a few caveats to the survey administration and the response rates, and we detail those in the limitations section. Lastly, Gibbs et al. (2022) document the details regarding the Institutional Review Board (IRB) and compliance across sites.

The TFS survey was the baseline survey, considered as Time 0, and was included as a pre-test in all analyses. The first follow-up survey (the SAFS) was administered in the spring of the first year (Time 1) to students who took the TFS as freshmen the previous fall. The second follow-up survey was administered to sophomore students in spring at the end of the second year (Time 2). The third, fourth, and fifth follow-up surveys, administered at the end of students’ third, fourth, and fifth years, are considered Times 3, 4, and 5, respectively. Once enrolled in the study, students were surveyed each spring regardless of whether or not they remained at the BUILD institution, had moved to another institution (e.g., entered graduate school), or were not in school (e.g., working).

The analytic data for the analyses were in long form with multiple time points for each student. The number of follow-up surveys for each student ranged from one follow-up (31.9% of students) to five follow-up surveys (3% students). Our sample includes 551 BUILD students and 9097 non-BUILD students (see Table 1) with missingness across variables ranging from 4 to 10%. We discuss more about how missing data was handled in the following sections.

Lastly, all co-authors are members of the CEC, which is responsible for the consortium-wide evaluation. Three of the co-authors are social scientists with backgrounds in STEMM education, one co-author is a biostatistician, and another co-author has expertise in epidemiology and gerontology. Our role as consortium-wide evaluators shaped the design of the study, focused on all sites and understanding intentions to pursue a science-related research career across the entire BUILD initiative. As outsiders to the details of the project, we are mindful of the implications and potential explanations we can provide to explain differences in results by site.

### 2.2. Outcome Variable: Intentions to Pursue a Science Career

The dependent variable is students’ responses regarding their intent to pursue a science-related research career across the three surveys—the TFS, SAFS, and CSS. Students were asked “*Will you pursue a science-related research career?*” The responses to this item were on a 5-point Likert scale of “definitely yes,” “possibly yes,” “uncertain,” “possibly no,” and “definitely no.” For our study, we collapsed the 5-level response scale to a binary variable coded as “1” for the two categories, “definitely yes” and “possibly yes,” and “0” for the three lower categories, “uncertain,” “possibly no,” and “definitely no.”

Several factors motivated this choice. First, the lower intent categories had small cell counts, which necessitated at least some collapsing. We next considered a range of ordinal variable modeling options. A common choice is the cumulative logit model. Cumulative logit models fit to the data yielded similar patterns of results as those presented here using the binary outcome. However, the cumulative logit model uses the proportional odds assumption, which states that the relationship between the predictor variables and the odds of being in a higher versus a lower category of the outcome variable is the same across all possible cutoffs of the outcome variable. In our application, this means assuming that the odds of “definitely yes” versus “possibly yes” or a lower category are equal to the odds of either “definitely yes” or “possibly yes” versus a lower category, or the odds of the top three versus the bottom two categories, etc., which is a strong assumption for our data setting. When the proportional odds assumption is violated, one can use multinomial logistic regression or partial proportional odds models. However, these models produce multiple sets of coefficients for each predictor, which adds considerable complexity to interpretation. Furthermore, the key focus of our study is to understand the relationship between students’ participation in BUILD and the outcome of intent to pursue rather than how *probable* students are to respond to “definitely yes” versus “possibly yes,” for example. In contrast, using a binary outcome yields a relatively straightforward interpretation that aligns with our study objectives without risk of violating model assumptions. Thus, we opted to use a binary outcome. Next, we compared models that dichotomized as “definitely yes” versus lower with models that dichotomized as “definitely yes” or “possibly yes” versus lower. The first option yielded sparse cells in some multivariable analyses, resulting in unstable estimates. Thus, we opted to dichotomize as the top two versus the bottom three categories.

### 2.3. BUILD Participation Variable

As noted above in the section on the BUILD initiative, we operationalized participation in BUILD to reflect students who were either BUILD Scholars or Associates. For the longitudinal analysis, we created a time-varying BUILD variable, which indicated whether a student had been admitted to the BUILD program as a Scholar or Associate as of the time that they took a given survey; that is, BUILD takes a value of “0” until a student is admitted to the program and remains “1” thereafter. This indicator variable remains “0” for students who were never admitted to the BUILD program, that is, non-BUILD students.

### 2.4. Background Characteristics Variables

We included the following student background characteristics in the analyses, race and ethnicity, gender, Pell grant awardee status, first-generation college student status, major at time of college entry (categorized as biomedical natural science, biomedical social science, or non-biomedical), high school grades, and financial worry about having sufficient funds for college (major, some, or none). Biomedical science majors include biology and zoology, for example, whereas biomedical social science includes psychology and sociology. These are student responses to items from the TFS survey.

### 2.5. Additional Explanatory Variables

We included additional variables such as students’ research experiences, scholarship received, and having a mentor as explanatory variables in the analysis. These variables were based on student survey responses and were coded as time invariant.

#### 2.5.1. Research Experience

The research experience variable is composed of student responses to the item in the CSS, “Since entering college, have you participated in an undergraduate research program,” and in the SAFS, “In the past 12 months, have you had any opportunity to conduct your own scientific research or to participate in scientific research directed by others?” This indicator variable takes on a value of “1” for all students who responded “Yes” to these items and “0” if the response is “No”. Additionally, students who indicated a non-zero number of months to the CSS item, “How many months since entering college (including summer) did you work on a professor’s research project?” (response options: 1 = 0 months, 2 = 1–3 months, 3 = 4–6 months, 4 = 7–12 months, 5 = 13–24 months, 6 = 25+ months) were coded as “1”.

#### 2.5.2. Mentoring

Information on whether a student had a mentor was derived from single items in the CSS and SAFS surveys. The CSS item is, “*Since entering college, have you found a faculty or staff mentor?*” and the SAFS item is “*Do you have a mentor?*” For our analyses, we used an indicator variable that takes on a value of “1” for all students who responded “Yes” to at least one of these items, and a value of “0” if the responses were “No.”

#### 2.5.3. Scholarships

Scholarships received was a single survey item, “*During the past 12 months, did you receive any scholarships or grants for education expenses that you do not need to repay?*” We used an indicator variable that takes on a value of “1” for all students who responded “Yes” to receiving scholarships, and a value of “0” if the responses were “No.”

### 2.6. Analyses

The analyses for the current study included three key steps. First, we conducted descriptive analyses comparing the BUILD and non-BUILD students on all background variables at baseline (Time 0). We calculated frequencies and percentages for categorical variables and mean and standard deviation for continuous variables. Differences between BUILD and non-BUILD students were tested using Chi-square and two-sample t-tests. Next, we tabulated the percentage of responses and Chi-square tests for BUILD and non-BUILD students over time with respect to the outcome of intention to pursue a science-related research career.

Secondly, we conducted the propensity score estimation as we have an observational study design. The student sample was based on participation in the BUILD program, resulting in non-equivalent groups of students: those participating in the program and those who were not participating. Given the potential for selection bias, which could confound the results, we used propensity score methods to create groups of students in treatment (participating in BUILD) and control (those who are not participating in BUILD) with similar distributions of all key covariates (see Table 1) (Rosenbaum & Rubin, 1983). Comparing outcomes between treated and control participants who share a similar value on the propensity score reduces the effects of confounding (Stuart, 2010). Additionally, for observational data, Austin (2011, p. 417) discusses the advantages of propensity score-based methods over regression adjustment-based methods when estimating treatment effects.

We conducted propensity score estimation and weighting using the MatchIt package (Ho et al., 2011) in R (R Core Team, 2022). For the propensity score estimation model, the outcome variable was a binary indicator variable of whether the student had “ever” participated in the BUILD program, and the covariates included were at baseline, that is, the covariates were measured prior to treatment (See Table 1 for a list of covariates). We included the intent to pursue a science-related research career item (binary)at baseline (Time 0) as a pre-test variable in the outcome model, and also students’ science identity at baseline. This measure was an item response theory (IRT) scaled score operationalized using four items^1^ from the SAFS on a 5-point scale ranging from “strongly disagree” to “strongly agree” (see Eagan et al., 2023) and is an important measure of how students identify as a scientist. We made use of the “full matching” approach wherein every student in the BUILD group (treatment) is matched to at least one non-BUILD student (control group), and every student in the control group is matched to at least one student in the treatment group (Stuart & Green, 2008). After acceptable balance (e.g., examining the quality of the match and balance tables) was achieved on baseline covariates, we proceeded to estimate the treatment effects.

Missing data across the outcome variable and the covariates were generally minimal, with about 7% missing responses with respect to our outcome variable of intentions to pursue a science-related research career. We had no missingness across gender, race/ethnicity, and high school GPA variables. Across financial worry, major, and first-generation status variables, there was 4 to 10% missingness. We made use of listwise deletion across the covariates to handle missingness among the covariates.

Finally, for our outcome models, we fit a series of mixed-effects logistic regression models wherein repeated measures over time are nested within students, and we included inverse propensity score weights. As noted by Joffe et al. (2004), regression models with inverse probability treatment weights can be used to estimate causal effects of treatments. We used inverse propensity weights to estimate the average effect of treatment on the treated (ATT) and the average treatment effect (ATE). The ATT is the effect of the treatment (program) on the population potentially exposed to the treatment, while the ATE estimates the potential effect of the treatment on the wider population (Guo & Fraser, 2014). This approach allowed us to examine the effectiveness of the BUILD initiative and study its impact on undergraduate students’ intentions to pursue a science-related research career among students receiving the program, and estimate its potential impact if it were implemented for a wider population of students.

In the four nested models, we included each variable in a stepwise fashion; this enabled us to examine the effect of BUILD and the contribution of the key components of BUILD. Model 1 included fixed effects for time and sites as well as indicators for BUILD, and we controlled for intent to pursue (binary) at Time 0. We included indicators for Time 2 (student responses at the end of the second year) through Time 6, with Time 1 (student responses at the end of the first year) as the reference group. We included a random intercept for the student. Next, in Model 2, we added research experience to examine changes to the coefficients. In Model 3, we added whether a student had a mentor, and in Model 4, we included whether the student received a scholarship. Missing data across these additional explanatory variables reduced our final analytic sample for the mixed-effects models with repeated measures for time to 12,940 observations and 6840 students.

## 3. Results

### 3.1. Sample Characteristics

Table 1 presents demographic information for the study sample at baseline (fall of students’ first year, Time 0), comparing undergraduates who subsequently participated in the BUILD program to those who did not. Students who subsequently participated in BUILD as Scholars or Associates (i.e., BUILD students) were more likely to indicate they intended to pursue a career in science as an entering first-year student (76.5% vs. 53.9% among non-BUILD students). BUILD students were also more likely to report a biomedical natural science major (82.5% vs. 68.7% among non-BUILD students) and higher science identity (average score 59.3 vs. 54.3). BUILD students reported lower levels of financial worries compared to non-BUILD students. BUILD students were similar to the non-BUILD students in terms of gender distribution (X^2^ [2, 9648] = 1.4937, *p* = 0.474), but were more likely to identify as Black/African American for their race/ethnicity (30.6% of BUILD students) and less likely to report as Hispanic/Latine (20.2% of BUILD students). BUILD students were less likely to report being first-generation college students (17.2% vs. 25.3%) and had higher GPAs (e.g., 36.1% A or A+ vs. 29.0%).

As students moved through their college years, both BUILD and non-BUILD students showed a decrease in the percentage reporting that they intended to pursue a science-related research career (see Table 2). However, the BUILD students were significantly more likely to report an intention to pursue a science-related research career compared to their non-BUILD counterparts (*p* < 0.001) for every year in college (Table 2). Additionally, we observed that the gap between BUILD and non-BUILD students’ intentions to pursue a science-related research career grew over time in college.

### 3.2. Propensity Score Estimation and Outcome Modeling

**Figure 1.**
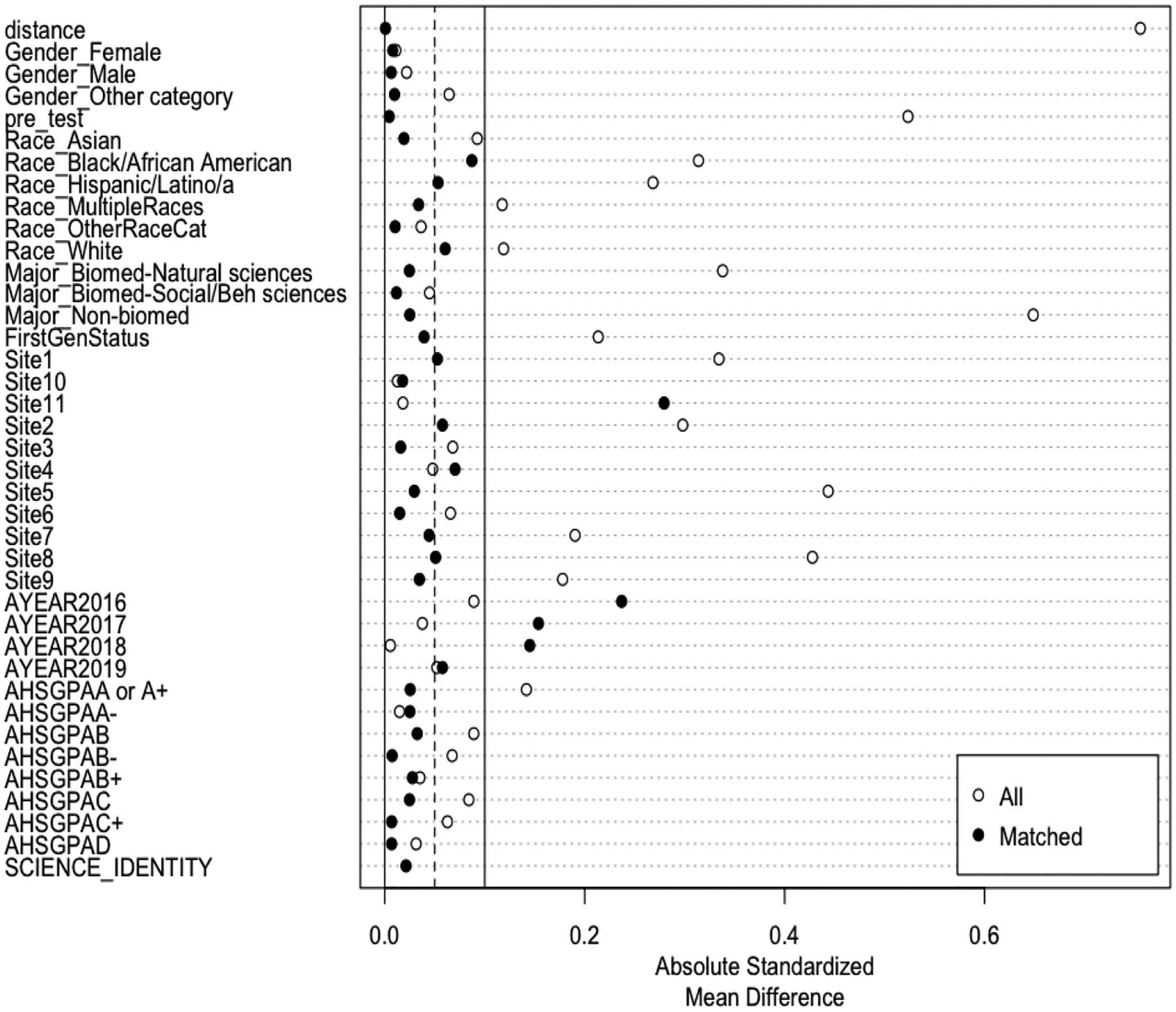
Balance depicted by the absolute standardized mean difference across all the covariates for ATT weighting.

**Figure 2.**
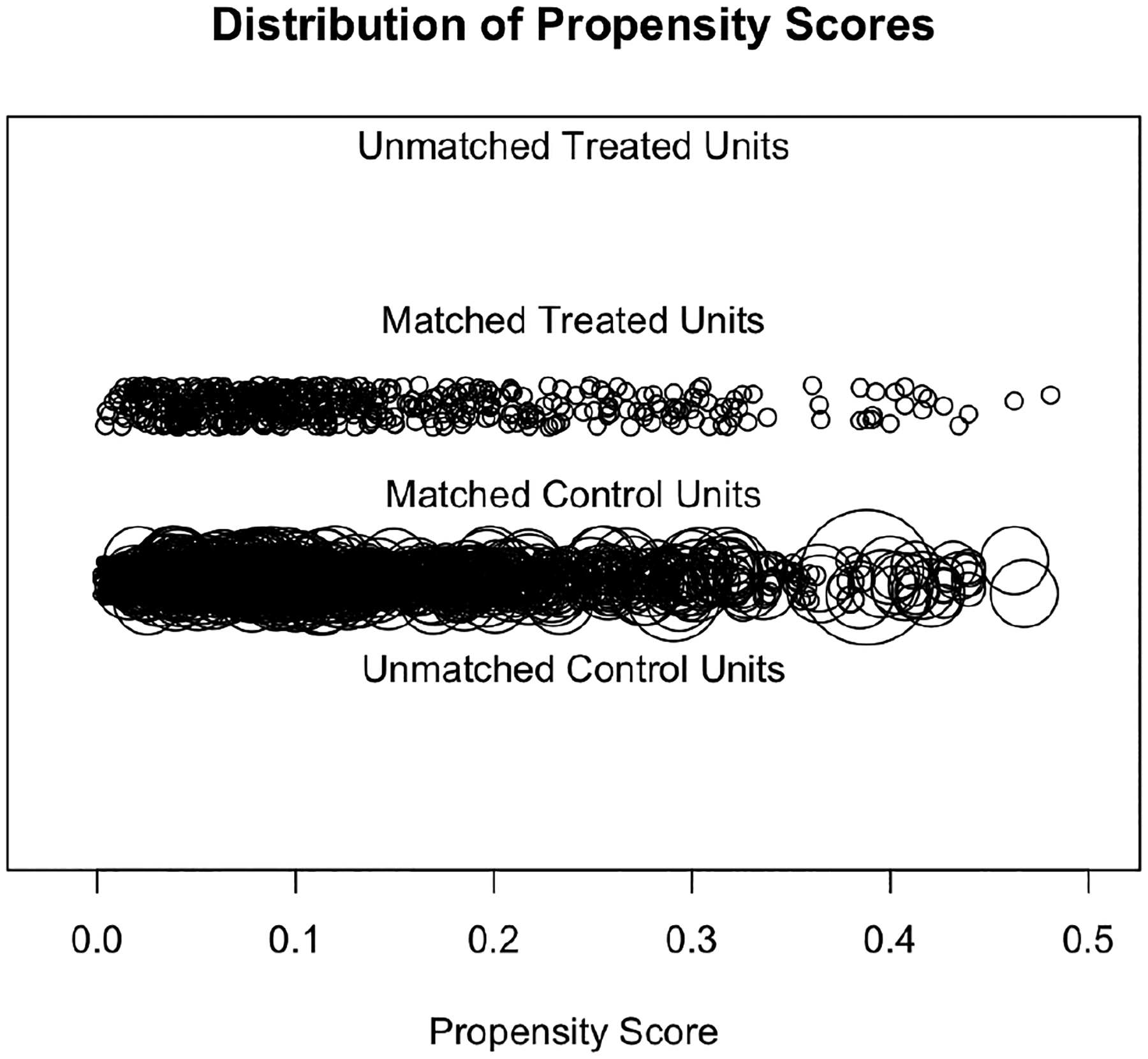
Distribution of the propensity scores for the ATT weighting.

Before fitting a series of mixed-effects logistic regression models to examine the effects of the BUILD program on students’ intentions to pursue a science-related research career, we examined the quality of the matching and ensured that the propensity score approach had effectively eliminated differences between the BUILD and non-BUILD students on observed covariates. Figure 1 visualizes the balance across covariates for the ATT weighting approach using the absolute standardized mean difference (SMD). The SMD is the difference in the means of each covariate between treatment groups, with regard to a standardization factor, so that it is on the same scale for all covariates. An absolute SMD close to a value of 0 indicates that we have good balance, and literature suggests absolute values be less than 0.1 (Greifer, 2022). As shown in Figure 1, we found substantial reductions in effect sizes (SMDs less than 0.1) post-weighting for most of the pre-treatment covariates, indicating that the pre-treatment differences between the BUILD and non-BUILD students were reduced or eliminated after weighting. Balance on cohort year (Year 2016, Year 2017, Year 2018, Year 2019 in Figure 1) was not achieved at the 0.1 benchmark for all years, which is a limitation. Next, in Figure 2, we visualized the distribution of propensity scores of those who were matched using a jitter plot. This plot indicates that there were no unmatched students. Similar checks were conducted for ATE weights as well.

#### 3.2.1. Measuring BUILD Effects Using ATE Weights

Table 3 shows the results of the mixed-effects logistic regression modeling with ATE weights, which estimates effects for the wider population from which the sample is drawn. The stepwise models examine the extent to which research experiences, having a mentor, and receiving scholarships account for the overall BUILD effect. In Model 1, after accounting for the sites as fixed effects, BUILD students had significantly stronger intentions to pursue a science-related research career compared to their non-BUILD counterparts (OR = 8.44). In the subsequent stepwise models, the BUILD effect attenuates as we add students’ self-reported responses of research experience, having a mentor, and scholarship received. However, even after accounting for such experiences (Model 4), BUILD students had four times the odds of reporting an intent to pursue a science-related research career (OR = 4.44 in Model 4) in comparison to undergraduates who did not participate in BUILD programs during any point in their college careers. Additionally, in our final model (Model 4), students with research experiences had twice the odds as those without research experiences to report science-related research career plans (OR = 2.38), and students who had a mentor had 1.32 times higher odds to pursue a science-related research career relative to students without mentors. All models showed a decrease in students’ intention to pursue a science-related research career as students moved through their college years. Sensitivity analysis that included control for cohort year in the mixed-effects models did not appreciably change the results.

Including undergraduate research experience, having a mentor, and receipt of scholarships to the models (Model 4) enabled us to capture whether non-BUILD students also reported engagement in these activities, which might have also been provided outside of the BUILD initiative. For example, some non-BUILD undergraduates may have obtained undergraduate research experience and reported having a mentor from involvement in a lab with a faculty member on campus who was not affiliated with BUILD.

#### 3.2.2. Measuring BUILD Effects Using the ATT Weights

Next, we examined the mixed-effects logistic regression models with ATT weights, which are the estimates for the average effect of the treatment on the treated population (see Table 4). The results in Table 4 indicate that the BUILD students continued to have significantly stronger intentions to pursue a science-related research career relative to their counterparts; that is, BUILD students had two times higher odds of having higher science-related research career intentions (OR = 2.36, *p* < 0.001) relative to non-BUILD students. In comparison to the ATE, the ATT estimand can be useful in situations when a treatment or program would only ever be administered to certain types of people and the focus would be only the group of people who fit that profile; we would be interested in the average effect of the treatment for people who would participate or be exposed to this treatment. The ATT effect estimates the effectiveness of treatment among individuals with higher probabilities of receiving the treatment. In other words, this model’s weighting aims to compare BUILD students to other undergraduates who had similar characteristics that would have made them strong BUILD candidates, but ultimately did not participate in BUILD. As such, we see slightly smaller BUILD effects in comparison to models with ATE weighting. In the final model (Model 4), BUILD students had one and a half times higher odds of pursuing a science-related research career relative to non-BUILD students. Having research experiences and a faculty mentor were significant predictors of students’ intention to pursue a science-related research career independently of BUILD exposure, and vice versa. Sensitivity analysis that included control for cohort year in the mixed-effects models did not appreciably change the results.

### 3.3. Limitations

There are several limitations of this study. First, due to the small number of sites (11 undergraduate institutions), we were unable to examine site-level characteristics. Future qualitative and mixed-methods studies can help us better understand the different BUILD programs across various sites and contextual factors associated with BUILD program efficacy. This site-level analysis will include various data sources that are currently being collected across sites (e.g., institutional records data, case studies) to capture the variation among students’ intentions to pursue a science career within the BUILD institutions from different geographical locations and institutional types. Furthermore, we believe that these additional data sources will provide nuanced participation information such as the total number of hours students participated in research experiences, if these experiences were on campus or off campus, and also the mentoring relationship between mentee and mentors for BUILD students (see Maccalla et al., 2022). Second, our study is a quasi-experimental design, which limits us in terms of the causal inferences we can draw, and while we have included a range of variables in our propensity score model to control for confounding, our results could still be confounded by unobserved variables. Third, not all eligible students participated in the surveys, which resulted in potential bias and smaller samples. For example, during the initial survey administration, at some sites, CEC capped the sample at 500, which could result in low response rates for sites that might have a large number of first-year students. Therefore, the exact response rates are difficult to know, and general estimates are provided.

## 4. Discussion

This study examined longitudinal data from all the 10 BUILD awarded sites (11 higher education institutions), including four cohorts of undergraduate students, to discern patterns regarding students’ intentions to pursue a science-related research career over time in college, with particular attention to the potential influence of activities developed or enhanced by the BUILD initiative. We present a summary of the results, as well as implications for education policy and practice.

### 4.1. Summary of Results

At college entry (first-year students), a larger percentage of students who would become BUILD participants later in their college career reported having strong intentions to pursue a science-related research career compared to students who were never involved with BUILD. Our results suggest an overall decrease in intentions to pursue a science-related research career for both BUILD and non-BUILD students over time in college, which is consistent with prior research. However, at each year in college, intentions among BUILD students were higher than those among non-BUILD students. Students entering BUILD programs often have higher GPAs and identify strongly as scientists compared to those who never became BUILD students, which could contribute to their higher intentions at each time point or year in college.

The propensity scoring approach enabled us to create similar groups of students for whom we could draw valid inferences regarding the impact of BUILD. The results with ATE weighting allow us to draw inferences regarding the full population of BUILD and non-BUILD students. Our findings suggest that after accounting for research and mentoring experiences, the effect of participation in BUILD remained sizable, with BUILD Undergraduates being four times more likely to pursue a science-related research career in comparison to their non-BUILD counterparts. These results suggest a benefit from BUILD exposure beyond that conferred by these experiences. Next, with ATT weighting, we continued to find significant BUILD effects; however, we found that BUILD Undergraduates were twice as likely to pursue a science-related research career after controlling for sites, baseline intent to pursue, research experience, having a mentor, and receipt of scholarships. Note that the results in Tables 3 and 4 speaks to the overall efficacy of BUILD programs and these results can be helpful in deciding the type of programs that must be developed by institutions and those which are most useful for students when there are limited resources, and more work is needed to understand the specific site-level contextual effects.

These findings, that the effect of BUILD remained significant even after adjusting for research experience, mentoring and receipt of scholarships, suggest that it is not just the separate components of BUILD but rather something about how these components are organized and presented to the students involved in BUILD that provides other benefits not accounted for in the regression model. The BUILD programs provide students with tuition support or a stipend, research training, undergraduate research experiences, a learning community, and mentorship. These various BUILD-related activities are supporting and contributing toward BUILD students’ intentions to pursue science-related research careers. While there is a great deal of previous research on the importance of undergraduate research to support STEMM career pathways (Carpi et al., 2017; Eagan et al., 2013; Hurtado et al., 2009), this study’s findings suggest that undergraduate research and mentoring are important over time for mitigating the loss of one’s desire to pursue a science-related research career.

### 4.2. Lessons Learned: Implications Related to STEMM Policy and Practice

An important contribution of this study is evidence of the importance of comprehensive STEMM training programs in maintaining students’ intentions to pursue a science-related research career, above and beyond key evidence-based college experiences in STEMM. BUILD differed from other training programs in that the awardees simultaneously focused on student-level, faculty-level, and institutional-level capacity-building. Considering that the BUILD initiative was an experimental award to collect data to help improve research training programs and inform the design of future programs so they can better develop talent from all backgrounds (Valantine et al., 2016), this study suggests that a multi-level approach that aims to transform the ecosystem for trainees is worth the investment. These can help policymakers make their decisions to fund such interventions, which can increase the proportion of students entering the scientific workforce. Overall, the impact of comprehensive and coherent programs can be stronger than the individual components and provide the much-needed psychological sense of community, and the shared lived experiences contribute vastly to positive academic and psychological outcomes.

Considering that students’ intentions to pursue a science-related research career diminish as they progress through college, and previous research suggested this for the underrepresented students (Fry et al., 2021), future work must examine if interventions such as BUILD can help to mitigate the diminishing attraction of pursuing a science career as students’ progress through college. We recommend that institutions invest in and dedicate their efforts to undergraduate research and strengthening STEMM faculty and peer mentoring programs. Particularly for institutions that might not have current National Science Foundation (NSF) or NIH training grants, institutional funds or collaboration from student affairs and academic affairs offices can be leveraged to develop and synergize mentoring efforts on a college campus. Several institutions nationwide are moving toward enhancing or creating undergraduate research offices in order to consolidate administrative efforts and structures to facilitate undergraduate research opportunities. Creating spaces which act as a central source of support for students when they are faced with unexpected challenges or barriers can help reduce the difficulty of continuing to pursue career intentions. The study’s findings not only underscore the longstanding social science evidence base on the importance of undergraduate research (Carpi et al., 2017; Eagan et al., 2013) and having a mentor (A. Byars-Winston & Dahlberg, 2019), but provides new evidence that these experiences are valuable whether they occur within a coordinated undergraduate scholar program such as BUILD, or even outside of participation in a comprehensive STEMM training program. It is important to note that the sample for this study provides a rich evidence base with a diverse sample of students from a diverse set of BUILD awardee sites. Incoming students at BUILD sites reflect the large proportion of students recognized as being underrepresented in the STEMM research workforce (Hurtado et al., 2017; Norris et al., 2020). For example, the sample consists of 17% who identify as Black/African American, 28% who identify as Hispanic/Latine, and 26% who are first-generation students (see Table 1). Therefore, while we see that BUILD has a strong positive impact on the overall intentions to pursue a science-related research career, a question yet to answer is, “does students’ intentions to pursue a scientific career differ for students from different sub-groups, and if there is a differential effect of BUILD for these sub-groups?”

This is a very important next step because, while 11% of the employed adults across the U.S. population identify as Black or African American, only 9% of them are in STEM occupations, and 6% are in life sciences; likewise, individuals who identify as Hispanic/Latine make up 17% of the working population and only 8% of all are STEM workers (Fry et al., 2021). These statistics continue to be worse for Black/African American and Hispanic/Latine students who are earning advanced degrees in STEM, especially those earning Ph.D. or other research doctorates. To this end, in 2021, a Pew Research Center report argued that the long-term prospects for achieving and sustaining overall population parity of traditionally underrepresented groups in the STEM workforce are closely tied to representation and participation in STEM at U.S. colleges and universities (Fry et al., 2021). Higher education institutions must support degree completion and career preparation for students from historically excluded groups and focus on enhancing participation in STEM.

Lastly, on the methodological front, for future work, we plan to conduct site-level analyses using various data sources (e.g., institutional records data, case studies) to capture the variation among students’ intentions to pursue a science career within the BUILD institutions from different geographical locations and institutional types. We plan to work in a multi-level modeling setting while employing appropriate adjustments, such as the Kenward–Roger adjustment (Kenward & Roger, 1997) and/or a Bayesian estimation approach (McNeish & Stapleton, 2016), to help guard against the inflated type-I error rate that can result from underestimated fixed-effect standard errors.

## 5. Conclusions

The evaluation of large federally funded multi-site programs can be challenging and complex. As the field moves toward developing more refined and empirically robust theories of action for best engaging students in STEMM majors, including those from background that are historically underrepresented, the evaluation design and analytic approach employed in this study offers a rigorous understanding of the relationship between students’ participation in activities that have been shown to matter and important outcomes of interest. Ideally, these efforts will shift the biomedical workforce to better reflect our nation’s diverse population. This study makes an important contribution in advancing our understanding of the effectiveness of large federally funded programs or investments, given that previous studies have often used a single-institution approach without comparison groups or quasi-experimental designs. Incorporating lessons learned from the DPC’s initiatives about mentoring and research training components, and about the comprehensive BUILD program, can greatly benefit the next generation of STEMM students by informing program leaders about what elements of their training programs are most effective while also helping institutions ensure that all students feel supported and safe in STEMM.

## Figures and Tables

**Table 1.** Descriptive statistics of all variables at baseline (Time 0) for non-BUILD and BUILD students.

Variable	Non-BUILD Students		BUILD Students		Chi-Square Tests
	(N = 9097)		(N = 551)		
	n	%	n	%	
Major					
Biomedical Natural Science Field	5863	68.7	435	82.5	X^2^ (2, 9648) = 75.34, *p* < 0.001
Biomedical social science field	933	10.9	66	12.5	
Non-biomedical field	1735	20.3	26	4.9	
Financial Worry					
Major (not sure I will have enough funds to complete college)	1610	19.2	97	18.4	X^2^ (2, 9648) = 10.344, *p* = 0.0056
Some (but I probably will have enough funds)	5133	61.1	295	56.1	
None (I am confident that I will have sufficient funds)	1657	19.7	134	25.5	
Gender					
Female	6152	67.7	371	67.3	X^2^ (2, 9648) = 1.4937, *p* = 0.474
Male	2844	31.3	177	32.1	
Non-binary/Other	97	1.1	3	0.5	
Race/Ethnicity					
Asian	1875	20.8	92	16.7	X^2^ (5, 9648) = 87.63, *p* < 0.001
Black/African American	1522	16.9	168	30.6	
Latine	2573	28.5	111	20.2	
Multiple races	651	7.2	60	10.9	
Other race category	188	2.1	13	2.4	
White	2216	24.6	106	19.3	
First-Generation Status					
Non-first-generation students	6033	74.7	423	82.8	X^2^ (1, 9648) = 16.696, *p* < 0.001
First-generation students	2040	25.3	88	17.2	
High School GPA					
A or A+	2625	29.0	198	36.1	X^2^ (7, 9648) = 21.866, *p* = 0.003
A−	2624	29.0	166	30.3	
B	1305	14.4	59	10.8	
B−	328	3.6	12	2.2	
B+	1986	21.9	108	19.7	
C	58	0.6	2	0.4	
C+	122	1.3	3	0.6	
D	7	0.1	3	0.5	
	n	Mean (SD)	n	Mean (SD)	
Science Identity	7855	54.3 (8.2)	508	59.3 (7.7)	t(584.06) = 13.136, *p* < 0.001

**Table 2.** Percentage of students’ intentions to pursue a science-related research career over time for non-BUILD and BUILD students and Chi-square tests.

Time	Student’s Intention to Pursue a Science-Related Career		Chi-Square Tests of Outcome Comparing Non-BUILD vs. BUILD Students
	Non-BUILD N (%)	BUILD N (%)	
0	4266 (53.9)	391 (76.5)	X^2^ (1, 9655) = 100.38, *p* < 0.001
1	3039 (58.4)	291 (79.1)	X^2^ (1, 6068) = 61.36, *p* < 0.001
2	2346 (52.8)	313 (81.3)	X^2^ (1, 4967) = 116.38, *p* < 0.001
3	1969 (47.4)	313 (74.9)	X^2^ (1, 4705) = 114.43, *p* < 0.001
4	1075 (42.2)	203 (71.5)	X^2^ (1, 2993) = 88.30, *p* < 0.001
5	495 (38.1)	120 (75.5)	X^2^ (1, 1509) = 81.39, *p* < 0.001
6	168 (37.4)	38 (62.3)	X^2^ (1, 528) = 13.81, *p* < 0.001

Note: Time 0 is the baseline.

**Table 3.** Odds ratio (standard error) for the mixed-effects logistic regression models for students’ intentions to pursue a science-related research career with ATE weights.

Variables in the Model	Odds Ratio (Standard Error)			
	Model 1	Model 2	Model 3	Model 4
(Intercept)	0.42 (1.09) ***	0.41 (1.09) ***	0.38 (1.09) ***	0.42 (1.10) ***
BUILD	8.44 (1.18) ***	5.59 (1.19) ***	5.09 (1.19) ***	4.44 (1.21) ***
Intent to Pursue at Baseline	9.48 (1.08) ***	8.65 (1.08) ***	8.52 (1.08) ***	8.51 (1.08) ***
Time (Ref: Time 1)				
Time 2	0.67 (1.06) ***	0.66 (1.07) ***	0.66 (1.07) ***	0.66 (1.07) ***
Time 3	0.44 (1.07) ***	0.41 (1.07) ***	0.40 (1.07) ***	0.41 (1.08) ***
Time 4	0.30 (1.08) ***	0.26 (1.08) ***	0.26 (1.09) ***	0.30 (1.11) ***
Time 5	0.22 (1.10) ***	0.21 (1.11) ***	0.21 (1.11) ***	0.22 (1.13) ***
Time 6	0.13 (1.16) ***	0.13 (1.17) ***	0.12 (1.17) ***	0.11 (1.19) ***
Site (Ref: Site1)				
Site 2	1.30 (1.14)	1.26 (1.14)	1.25 (1.14)	1.21 (1.15)
Site 3	2.29 (1.18) ***	2.00 (1.19) ***	2.10 (1.19) ***	2.14 (1.19) ***
Site 4	0.97 (1.13)	0.94 (1.13)	0.90 (1.13)	0.88 (1.13)
Site 5	1.87 (1.15) ***	1.74 (1.15) ***	1.74 (1.15) ***	1.70 (1.15) ***
Site 6	1.26 (1.17)	1.22 (1.17)	1.19 (1.17)	1.19 (1.17)
Site 7	1.14 (1.16)	0.99 (1.17)	0.94 (1.17)	0.92 (1.18)
Site 8	1.42 (1.10) ***	1.27 (1.10) *	1.24 (1.10) *	1.16 (1.10)
Site 9	2.30 (1.14) ***	2.07 (1.14) ***	2.10 (1.14) ***	2.05 (1.14) ***
Site 10	1.74 (1.16) ***	1.58 (1.17) **	1.60 (1.17) **	1.56 (1.17) **
Site 11	2.70 (1.14) ***	2.28 (1.14) ***	2.19 (1.14) ***	2.35 (1.15) ***
Research Experience		2.29 (1.07) ***	2.16 (1.07) ***	2.38 (1.08) ***
Have a Mentor			1.36 (1.06) ***	1.32 (1.06) ***
Scholarship Received				0.90 (1.06)

Notes: BUILD indicates scholars and associates and is time-varying.

* *p* < 0.05.

** *p* < 0.01.

*** *p* < 0.001.

**Table 4.** Odds ratio (standard error) for the mixed-effects logistic regression models for students’ intentions to pursue a science-related research career with ATT weights.

Variables in the Model	Odds Ratio (Standard Error)			
	Model 1	Model 2	Model 3	Model 4
(Intercept)	1.65 (1.21) **	1.60 (1.21) *	1.38 (1.22) ***	1.63 (1.25) *
BUILD	2.36 (1.14) ***	1.80 (1.14) ***	1.65 (1.14) ***	1.56 (1.16) **
Intent to Pursue at Baseline	7.66 (1.13) ***	7.17 (1.13) ***	6.94 (1.13) ***	7.56 (1.15) ***
Time (Ref: Time 1)				
Time 2	0.49 (1.09) ***	0.45 (1.10) ***	0.42 (1.10) ***	0.39 (1.11) ***
Time 3	0.25 (1.10) ***	0.20 (1.10) ***	0.19 (1.11) ***	0.17 (1.12) ***
Time 4	0.15 (1.11) ***	0.11 (1.12) ***	0.10 (1.12) ***	0.13 (1.16) ***
Time 5	0.09 (1.14) ***	0.08 (1.14) ***	0.07 (1.15) ***	0.08 (1.17) ***
Time 6	0.05 (1.19) ***	0.04 (1.20) ***	0.04 (1.20) *	0.04 (1.24) ***
Site (Ref: Site1)				
Site 2	3.60 (1.56) **	3.36 (1.57) **	3.18 (1.59)	3.13 (1.70) *
Site 3	1.83 (1.37)	1.56 (1.38)	1.69 (1.38)	2.18 (1.44) *
Site 4	0.97 (1.24)	1.03 (1.25)	0.99 (1.25)	1.08 (1.28)
Site 5	3.06 (1.67) *	2.47 (1.71)	2.75 (1.75)	3.06 (1.83)
Site 6	1.53 (1.35)	1.72 (1.36)	1.66 (1.36) **	1.64 (1.40)
Site 7	0.61 (1.23) *	0.57 (1.23) **	0.54 (1.24)	0.48 (1.26) **
Site 8	0.88 (1.18)	0.85 (1.18)	0.85 (1.19) **	0.78 (1.20)
Site 9	2.41 (1.32) **	2.41 (1.33) **	2.43 (1.33)	2.46 (1.38) **
Site 10	1.44 (1.28)	1.38 (1.29)	1.44 (1.29) **	1.57 (1.32)
Site 11	2.44 (1.23) ***	2.06 (1.24) ***	2.04 (1.24) ***	2.00 (1.27) **
Research Experience		2.54 (1.08) ***	2.26 (1.09) ***	2.94 (1.1) ***
Have a Mentor			1.79 (1.08)	1.77 (1.09) ***
Scholarship Received				0.75 (1.10) **

Notes. BUILD indicates scholars and associates and is time-varying.

* *p* < 0.05.

** *p* < 0.01.

*** *p* < 0.001.

## Data Availability

Access to the data used for this paper can be granted to researchers completing and submitting an application for a data request. Forthcoming, in 2026, data from the Enhance Diversity Study (EDS) will be publicly available for request at the University of Michigan’s ICPSR data repository.

## Footnotes

1 The science identity scale is operationalized using four agreement items: I have a strong sense of belonging to a community of scientists; I derive great personal satisfaction from working on a team that is doing important research; I think of myself as a scientist; and I feel like I belong in the field of science (Estrada et al., 2011).
